# Lymphatic Metastasis of Esophageal Squamous Cell Carcinoma: The Role of NRF2 and Therapeutic Strategies

**DOI:** 10.3390/cancers17111853

**Published:** 2025-05-31

**Authors:** Yahui Li, Zachary Ladd, Zhaohui Xiong, Candice Bui-Linh, Chorlada Paiboonrungruang, Boopathi Subramaniyan, Huan Li, Haining Wang, Curt Balch, David D. Shersher, Francis Spitz, Xiaoxin Chen

**Affiliations:** 1 Surgical Research Lab, Department of Surgery, Cooper University Health Care, Camden, NJ 08103, USA; li-yahui@cooperhealth.edu (Y.L.); laddza45@rowan.edu (Z.L.); subramaniyan-boopath@cooperhealth.edu (B.S.); li-huan@cooperhealth.edu (H.L.); shersher-david@cooperhealth.edu (D.D.S.); spitz-francis@cooperhealth.edu (F.S.); 2 Cooper Medical School of Rowan University, Camden, NJ 08103, USA; 3 Coriell Institute for Medical Research, Camden, NJ 08103, USA; zxiong@coriell.org (Z.X.); cbui-linh@coriell.org (C.B.-L.); cpaiboonrungruang@coriell.org (C.P.); cbalch@coriell.org (C.B.); 4 Insilico Medicine Canada Inc., Montreal, QC H3B 4W8, Canada; haining.wang@insilicomedicine.com; 5 MD Anderson Cancer Center at Cooper, Camden, NJ 08103, USA

**Keywords:** esophageal squamous cell carcinoma, lymphatic metastasis, NRF2

## Abstract

The lethality of esophageal squamous cell carcinoma (ESCC), and other epithelial cancers, is primarily due to its aggressive nature and frequent lymphatic metastasis, both of which impact prognosis. In this review, we explore the underlying molecular mechanisms of ESCC lymphatic metastasis, specifically, the functional role of NRF2 and therapeutic strategies. Current data suggest that NRF2 hyperactivation (NRF2^high^) may promote lymphatic metastasis of ESCC by affecting the extracellular matrix (ECM), epithelial–mesenchymal transition (EMT), lymphangiogenesis, immune evasion, metabolic programming, and Hippo signaling. We also update the latest developments in NRF2 inhibitors, their mechanisms of action, screening strategies, and approaches for evaluating compound efficacy. Finally, we highlight the utility of animal models for mechanistic studies and therapeutic development. We believe elucidation of the functional role of NRF2 in ESCC lymphatic metastasis and developing proper NRF2 inhibitors will greatly improve the clinical prognosis of ESCC in human patients.

## Simple Summary

Esophageal squamous cell carcinoma is a deadly cancer largely because it spreads quickly through the body’s lymphatic system. This review looks at how NRF2 might be involved in helping the cancer spread. When this protein is overly active, it may support cancer growth by influencing how cancer cells move, how they avoid the immune system, and how they change their environment. We also discuss current efforts to develop drugs that can block NRF2 activity and how to test these treatments in lab models. Understanding the role of NRF2 in this type of cancer and finding ways to target it could lead to new treatment strategies and improve outcomes for patients.

## 1. Introduction

Esophageal cancer is the seventh-most common, and sixth-most lethal, cancer, with over 604,100 new cases (3.1% of all cancer diagnosis) and 544,076 deaths (5.5% of all cancer-related deaths) in 2020 [1]. Specifically, esophageal squamous cell carcinoma (ESCC), one of two major histological types, has a 5-year survival rate of ~20% [2,3], with over 40% of patients already having lymph node metastasis upon ESCC diagnosis. Moreover, the presence of metastatic lymph nodes, number of involved nodes, and lymph node ratio are negative prognostic factors after esophagectomy [4,5,6,7]. Although the 5-year survival rate of localized ESCC is 48%, that number drops to 28% upon metastasis into surrounding tissues and/or regional lymph nodes, and to 5% in patients with distant organ metastasis [8].

**Figure 1:**
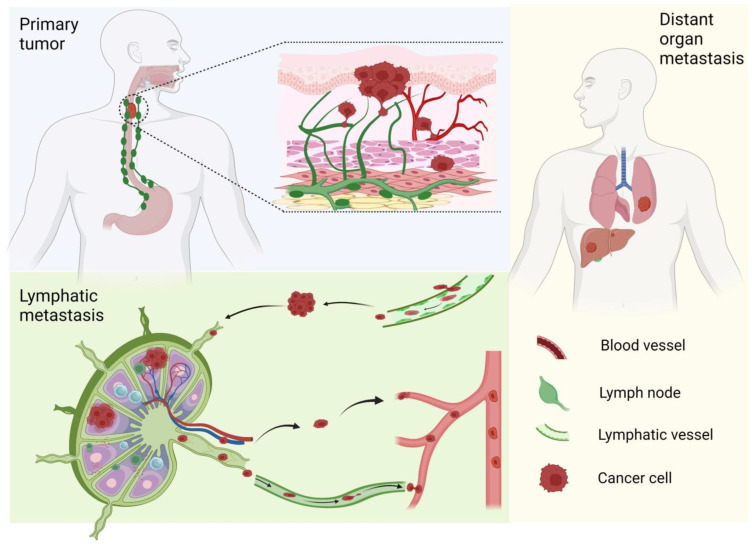
Clinical stages of metastatic ESCC. Primary tumors originate from esophageal epithelial cells. Once invading into the stroma, these cancer cells may penetrate lymphatic vessels and enter lymphatic circulation, and, finally, seed distant organs.

Similar to other epithelial malignancies, ESCC typically spreads through the lymphatic system to nearby draining lymph nodes [9]. This process begins with tumor cells breaching the basement membrane and progressively infiltrating deeper tissue layers, including the lamina propria, muscularis mucosae, submucosa, muscularis propria, and adventitia. Lesions extending up to the muscularis mucosae or slightly infiltrating the submucosa (up to 200 μm) are associated with an elevated risk of lymph node metastasis. About 50% of the lesions that show deeper (>200 μm) invasion into the submucosa are associated with metastasis [10]. Generally, the deeper the tumor penetrates the esophageal wall, the greater the risk of lymphatic spread [10,11]. Lymphangiogenesis represents an important prognostic marker for metastatic risk and overall survival, comprising a “premetastatic niche” (lymphatic vessels and lymph node) that facilitates cancer cell migration [12]. Further metastasis occurs through the bloodstream, colonizing distant organs (most commonly the lungs, liver, and bones) (Figure 1). It is therefore critical to understand how ESCC cells migrate, at each step, to develop better diagnostic and therapeutic strategies.

## 2. ESCC Lymphatic Metastasis

In the human esophagus, lymphatic vessels are located throughout the lamina propria, muscularis mucosae, submucosa, and muscularis propria. These structures not only facilitate lymphatic drainage, but may also serve as essential components of the stem cell niche, similar to their role in the skin [13]. The lymphatic vessels run in various orientations—longitudinally, transversely, and perpendicularly—and connect either directly to the thoracic duct or through a series of lymph node relays [14,15,16,17]. Regionally, lymphatic drainage differs along the esophagus, as follows: the upper third primarily drains into deep cervical lymph nodes and the thoracic duct; the middle third tends to drain into superior and posterior mediastinal lymph nodes; and the lower third drains into gastric and celiac lymph nodes. Importantly, these three lymphatic drainage zones are extensively interconnected [17,18,19].

In recent decades, clinical studies have shown several interesting features of ESCC metastasis, including the following: (1) Metastasis can take place when the primary tumor appears at an early stage. The abundant lymphatic network in the lamina propria may allow lymphatic metastasis to occur as early as Stage T1a, when tumor cells invade the lamina propria or muscularis mucosae. (2) There is a strong association between the depth of ESCC invasion and the likelihood of lymphatic metastasis. While Stage Tis—where cancer remains confined to the epithelial layer—typically does not involve lymphatic invasion or lymph node spread, the risk increases significantly in more advanced stages. Stage T1 tumors, which extend into the lamina propria or submucosa, and Stage T2 tumors, which infiltrate the muscularis propria, are more frequently linked to lymphatic dissemination [20,21,22]. (3) ESCC often exhibits complex metastatic patterns, including retrograde, bidirectional, and skip metastases [17,18,19,23]. Of 1074 ESCC patients with N1 disease (one or two positive lymph nodes), the majority of lymph node metastases occurred in the longitudinal direction to the peri-gastric region and bilateral recurrent nerve regions, and in the transverse direction, to the para-esophageal region [24]. As a result, sentinel lymph node biopsy or resection may be of limited value [25]. (4) The risk of lymphatic metastasis is associated with the location of the primary tumor, and cancer in the lower esophagus is more likely to metastasize to the lymph nodes than cancer in the middle or upper esophagus [26,27,28,29]. High lymphatic vessel density in the abdominal esophagus, especially the gastroesophageal junction, may be a contributing factor [30]. (5) Clinical metastases may not manifest until months or even decades following the initial diagnosis and/or tumor resection. Staging modalities, e.g., CT scan or endoscopic ultrasound, are of limited value for the detection of lymph node metastasis, and nodal micro-metastasis is undetectable by diagnostic procedures before surgery.

The clinical significance of lymph node metastasis in treating ESCC has been debated for decades [31]. For example, three-field (cervical–thoracic–abdominal) lymph node dissection during esophagectomy aims for complete regional dissection. In theory, extensive lymphadenectomy increases the likelihood of complete removal of all tumor-positive lymph nodes (including occult metastases). Indeed, three-field dissection was reported to significantly improve 5-year survival [32,33,34,35]. However, several controlled studies indicated that extensive lymphadenectomy during esophagectomy did not improve survival outcomes, and may increase the risk of postoperative complications [36,37,38], suggesting that lymph node metastasis may indicate systemic disease, rather than a direct determinant of survival. Rerouting of the lymph after lymphadenectomy may defeat the purpose of resection. The chaotic and unpredictable nature of ESCC lymphatic metastasis, and the limited impact of extensive lymphadenectomy, challenges current clinical guidelines, calling for further research to improve clinical practices.

## 3. NRF2 Signaling Pathway and Metastasis

The transcription factor nuclear factor erythroid 2-related factor 2 (NFE2L2 or NRF2), a well-known regulator of antioxidant response, has recently been identified to have multiple (non-antioxidative) roles [39]. Under normal physiological conditions, NRF2 activity is tightly regulated by its binding partner, KEAP1 (Kelch-like ECH-associated protein 1), which sequesters NRF2 in the cytoplasm and promotes its degradation via the ubiquitin–proteasome system. In addition to the KEAP1-CUL3-RBX1 complex, NRF2 is also regulated at the protein level by the other two E3 ubiquitin ligase complexes, HRD1 and SCF-βTrCP [40]. When NRF2 becomes hyperactivated, it drives increased cellular proliferation, metabolic reprogramming, enhanced metastatic potential, and resistance to various therapies, including immune checkpoint inhibitors, chemotherapy, and radiation [41]. In fact, persistent NRF2 activation has been implicated in the regulation of nearly all major hallmarks of cancer [39,42]. Five mechanisms are known to activate NRF2, such as somatic mutations of KEAP1/NRF2/CUL3, accumulation of disruptor proteins, skipping of NRF2 exon 2, KEAP1 succinylation, KEAP1 hypermethylation, electrophilic attack of KEAP1 by oncometabolites, and NRF2 overexpression through other mechanisms [39,43].

**Figure 2:**
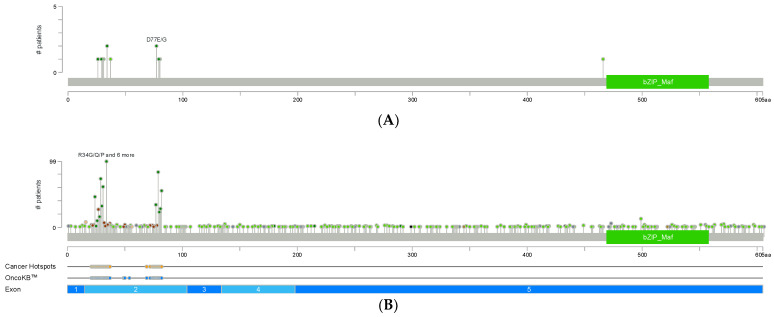
NRF2 mutations in human cancers and ESCC. (**A**) *NRF2* mutations in 227 samples in 2 ESCC studies. (**B**) *NRF2* mutations in 101,679 samples of 100,611 patients in 11 pan-cancer studies. Most frequent mutations of the DLG motif take place at the residue of R34 (R34G/R34Q/R34P/R34L/R34*/E35Kfs*6/R34_F37del/R34_K53del/R34_V36dup) and most frequent mutations of the ETGE motif at the residue of E79 (E79Q/E79K/E79V/E79D/E79G/E79A/X79_splice/E79L/E79dup). Graphs are downloaded from www.cbioportal.org. Two mutation hotspots (aa21-42 and aa73-82) overlap with the DLG motif (DEETGEFL, aa24-30) and ETGE motif (ETGE, aa79-82) of NRF2 protein, respectively, which are critical for its binding with KEAP1.

*NRF2* mutation is commonly seen in human ESCC [44,45]. Mutations in other genes of the NRF2 signaling pathway (*KEAP1* and *CUL3*) are present but less common than those in *NRF2*. *NRF2* mutations mainly occur in hotspots localized to the DLG and ETGE motifs which are required for its association with KEAP1 [46]. According to two sequencing studies on 227 cases of ESCC, *NRF2*, *KEAP1*, and *CUL3* mutation took place in 5.28%, 2.8%, and 0.9% cases, respectively (Figure 2A). In an integrated dataset that consists of 1930 ESCC genomes from 33 datasets, *NRF2*, *KEAP1*, and *CUL3* mutation took place in 7.62%, 2.75% and 2.49% cases, respectively. D29H and E79Q mutations are the most frequent ones in the DLG and ETGE motifs [47]. Depending on the patient population, the frequency of *NRF2* and *KEAP1* mutations can be as high as 35.9% and 4.1%, respectively [48]. Not only ESCC, but many other cancers also carry similar mutations. According to eleven pan-cancer sequencing studies on 101,679 samples from 100,611 patients, *NRF2*, *KEAP1*, and *CUL3* mutation took place in 1.8%, 4%, and 1.3% cases, respectively (Figure 2B). Similarly, in a Japanese cohort of 60,056 cancer patients, 1.7% and 2.5% carried mutations of *NRF2* (exon 2) and *KEAP1*, respectively [48].

The NRF2 signaling pathway plays a dual role in cancer development. On one hand, activation of NRF2, either genetically or through chemical inducers, stimulates the expression of cytoprotective enzymes that defend against chemical-induced tumorigenesis across multiple cancer types, including ESCC. On the other hand, persistent NRF2 activation supports tumor progression by reducing cellular stress from altered metabolism, hypoxia, immune surveillance, and uncontrolled proliferation. In ESCC, NRF2 overexpression is strongly associated with lymph node metastasis, tumor recurrence following surgery, and poorer overall survival outcomes [49,50,51,52]. As such, NRF2 is a well-established cancer driver gene and therapeutic target for ESCC [53]. A recent study analyzed the whole genomic, epigenomic, transcriptomic, and proteomic data of 155 cases of ESCC and led to the classification of the following four subtypes: cell cycle pathway activation (25.2%), NRF2 oncogenic activation (24.5%), immune suppression (19.3%), and immune modulation (31.0%) [54].

Many studies support NRF2′s role in promoting cancer metastasis. NRF2 activation has been shown to promote metastasis of melanoma and lung cancer [55,56,57]. In preclinical studies, two common type 2 diabetes mellitus drugs (saxagliptin and sitagliptin), which prolong the NRF2 antioxidant response by inhibiting KEAP1C151 (a cysteine sensor causing constitutive NRF2 activation), increase the metastatic risk of several cancers. NRF2 activation further upregulates metastasis-associated proteins, enhancing cell migration, and promoting metastasis in mouse xenograft models. Upon *NRF2* knockdown, both naturally occurring and saxagliptin- or sitagliptin-induced tumor metastasis were attenuated, whereas NRF2 activation accelerated metastasis. In human liver cancer tissues, increased expression of NRF2 and GCLM (a canonical NRF2 target gene) is associated with lymph node metastasis [58]. Implantation of cisplatin-resistant head and neck cancer cells into the tongues of nude mice resulted in a markedly elevated incidence of distant metastases. These drug-resistant cells exhibited strong upregulation of genes linked to the NRF2 signaling pathway, driven either by the newly emerged *KEAP1* mutations or by epigenetic activation of NRF2 target genes. Suppressing NRF2 expression or reintroducing *KEAP1^WT^* restored cisplatin sensitivity and reduced the extent of metastasis. Similarly, inhibition of glutaminase-1, a downstream gene regulated by NRF2, also mitigated resistance to cisplatin [59]. In breast cancer cells, increased NRF2 levels enhanced both proliferation and migration by upregulating key components of the pentose phosphate pathway (e.g., G6PD), including hypoxia-inducible factor 1α (HIF1α) and NOTCH1, with NOTCH signaling inducing breast cancer cell migration by upregulating the EMT [60].

On the other hand, the involvement of NRF2 in cancer metastasis remains controversial. NRF2 deficiency has been linked to increased metastasis in pancreatic cancer [61]. Similarly, *KEAP1* knockdown has varying effects depending on cancer type, for example, it inhibits metastasis in N87 (gastric cancer) and HCC1954 (breast cancer) cells, but enhances metastasis in HCC1806 and MCF7 (breast cancer) cells, with no impact on ZR-75-1 (breast cancer) cells [62]. One potential explanation for NRF2′s metastasis-suppressing role is that NRF2 activation may enhance cell survival by lowering oxidative stress, restricting partial EMT, and preventing tumor budding. This mechanism is believed to operate through the H₂O₂-dependent p38-MYC signaling pathway, which helps anchor tumor cells at the primary site [62].

NRF2 in cancer cells and host cells may also function differently in metastasis. *Nrf2^−/−^* mice display a higher incidence of pulmonary metastatic nodules, compared to their wild-type counterparts, following inoculation with mouse lung cancer cells. These cancer-bearing *Nrf2^−/−^* mice exhibited elevated numbers of inflammatory cells, including myeloid-derived suppressor cells, in both the lungs and bone marrow. Wild-type recipient mice transplanted with *Nrf2^−/−^* bone marrow cells exhibited increased lung metastasis after cancer cell inoculation, with significant accumulation of reactive oxygen species (ROS), correlating closely with decreased splenic CD8^+^ T cells. Conversely, *Keap1*-knockdown mice, characterized by NRF2 overexpression, underwent decreased lung metastasis, with reduced ROS within myeloid-derived suppressor cells [63]. Likewise, mice deficient for *Nrf2* in the myeloid lineage were increasingly susceptible to pulmonary metastasis from mouse lung cancer cells. Deletion of selenocysteine-tRNA, a gene crucial for synthesizing antioxidant selenoenzymes in the myeloid lineage, increased numbers of metastatic nodules and increased ROS in myeloid-derived suppressor cells in tumor-bearing mice. In addition, a synthetic NRF2 activator reduced ROS in myeloid-derived suppressor cells, consequently decreasing lung metastasis [64].

The mechanisms underlying the seemingly contradictory effects of NRF2 on cancer metastasis remain unclear. Consequently, the therapeutic impact of NRF2 inhibition may be either beneficial or detrimental, depending on factors such as cancer type, disease stage, NRF2 activity levels, and the tumor microenvironment. Although it is widely believed that ESCC cells can hijack the NRF2 pathway as a survival mechanism—and that NRF2 inhibitors may offer a therapeutic benefit in ESCC cases with elevated NRF2 activity—rigorous, context-specific studies are still needed to validate this approach in preclinical and clinical settings.

### NRF2 and the Cascade of Lymphatic Metastasis (Figure 3)

Lymphatic metastasis starts with epithelial cells undergoing the EMT, loss of polarity, and downregulation of cell adhesion molecules, disrupting cell–cell connections and enhancing migration and invasion of surrounding tissues. A minority of cells released from a tumor ultimately form distant lesions upon gaining multiple phenotypic traits that enable them to endure various stresses and become proficient at initiating the metastatic process. This dissemination can persist until the source tumor is removed [65].

ESCC cells exploit the mechanical forces and flow dynamics of both the blood and lymphatic systems to initiate metastasis. This process involves key steps such as intravasation and the release of tumor-derived signaling molecules from the primary site via the lymphatic network [66]. Once inside lymphatic vessels, tumor cells migrate through the lymph and respond to molecular signals that guide them to lymph nodes, where metastases can form. The presence of lymphoid cells at the primary tumor site has been strongly linked to increased lymphatic invasion. Furthermore, tumor-derived exosomes have been shown to enhance the expression of host genes that support lymph node colonization and tumor progression [67]. At the tumor invasion front, metastatic dissemination is driven by a combination of aggressive cell migration, remodeling of the ECM, and increased vascular permeability. These factors enable cancer cells to spread via the bloodstream, lymphatic vessels, perineural and perivascular pathways, or through direct invasion into adjacent body cavities [68].

As tumor cells navigate through the network of collecting lymphatic vessels, they eventually reach tumor-draining lymph nodes, which play a pivotal role in regulating antitumor immune responses [12,69]. In mouse models, the overexpression of VEGF-A during chemically-induced skin carcinogenesis led to enhanced lymphangiogenesis in metastatic tumor-draining lymph nodes. Similarly, mice with elevated VEGF-C expression in the skin exhibited increased lymph node lymphangiogenesis, which facilitated further metastasis to additional lymph nodes and distant organs [70].

Within lymph nodes—an environment rich in fatty acids—cancer cells typically first establish themselves in the cortical region after entering via the lymphatic circulation through the subcapsular sinus. Tumor cells release fibrogenic signals that activate mesenchymal cells in the surrounding stroma, leading to the production of a dense desmoplastic matrix. This matrix not only supports cancer cell invasion, but also promotes their proliferation by modulating mechanical signaling pathways [71,72,73,74]. Cancer cells adapt to the lymphatic environment selectively and dynamically, by adjusting their metabolism at every step during the metastatic cascade [75], shaping interactions with the host immune system by controlling the infiltration and reactivity of immune cells [12]. Nevertheless, cancer cell dormancy in lymph nodes is another option, as the host stroma presents physical, metabolic, and immune barriers that prevent cell growth [76].

Cancer cells can leave lymph nodes through the following two main pathways: (1) direct entry into the bloodstream via blood vessels within the lymph node and (2) continued migration through the lymphatic system. Blood vessels in lymph nodes serve as key conduits, enabling lymph-borne cancer cells to access the systemic circulation, potentially providing a more efficient route than direct spread from the primary tumor through blood vessels [77,78]. Because lymphatic vessels have lower flow rates and reduced shear stress compared to blood vessels, this lymphatic route may offer a less hostile environment for cancer cells during the initial stages of metastasis [79]. Cancer cells in the lymph are also better protected from ferroptosis, a form of cell death that is dependent upon iron-based lipid peroxidation, than cells in the blood, due to the abundance of fatty acids and antioxidants in the lymph [66,80].

In human ESCC, the genetic mutations found in metastatic lymph nodes differ from those in primary tumors [81]. Studies analyzing the “open” chromatin marker H3K27ac have identified active enhancers. Transcriptome analysis comparing primary tumors, metastatic lymph nodes, and nearby healthy esophageal tissue revealed thousands of enhancers that were either gained or lost, along with hundreds of altered potential super-enhancers in tumors and metastatic lymph nodes compared to normal tissue. Many of these changes are unique to metastatic lymph nodes [82]. Single-cell sequencing of these lymph nodes demonstrated that the metastatic microenvironment exhibited the appearance or growth of interferon-induced IFIT3^+^ T cells, B cells, and immunosuppressive cells (such as APOC1^+^APOE^+^ macrophages and myofibroblasts) that highly express immunoglobulin genes, ECM components, and matrix metallopeptidase genes [83].

NRF2 expression is important for migration of normal and malignant cells, as shown by *NRF2* knockdown, which greatly impairs migration and the invasion of a variety of cell lines. To facilitate migration, cells release ECM remodeling enzymes, including MMP2 and MMP9, which simultaneously release growth factors and cytokines entrapped within the ECM. Conversely, decreased NRF2 expression is associated with diminished MMP2 and MMP9 expression or gelatinase activity [84].

During migration and circulation, metastatic cells must overcome anoikis, i.e., a cellular death mechanism that occurs upon long-term detachment from the ECM. Genome-wide gene expression profiling has demonstrated that mutant NRF2 has a significant impact on various molecular pathways, including the survival-promoting mTOR pathway. Cancer cells, having persistently elevated NRF2, can proliferate in an anchorage-independent manner, consequently exhibiting increased metastatic potential [85]. Likewise, NRF2 activation induces the expression of protective genes (e.g., glyoxalase 1, which metabolizes methylglyoxal that adducts integrins), thus preventing anoikis [86].

The onset of metastasis requires a cellular process, the EMT, followed by invasion, transit, and eventually mesenchymal-to-epithelial transition, at a distant site. During EMT, epithelial cells lose expression of the adhesion protein E-cadherin and gain N-cadherin. In cervical cancer, NRF2 activation positively correlates with EMT and promotes both pulmonary and lymphatic distant metastasis in xenograft models. Likewise, a rescue experiment showed that NRF2 promoted metastasis partially through the EMT effector Snail1 [87]. In both bladder cancer and non-small cell lung cancer cells, NRF2 stabilized a hybrid EMT state, inhibiting complete EMT during collective cancer migration by upregulating DLL4 and JAG1 expression at the cells’ leading edge, correlating with leader cell formation. By contrast, NRF2 knockout compromised the hybrid E/M phenotype’s stability, which was reversible by NRF2 overexpression. Clinical data also corroborated a link between a hybrid E/M phenotype, elevated NRF2, and poor survival, emphasizing their significance in metastasis [88,89].

NRF2 deletion hinders the proliferation and metastasis of breast cancer cells by downregulating the cytoskeleton-binding GTPase RhoA while restoring RhoA counteracted growth and metastasis suppression in vitro. Additionally, NRF2 silencing diminished stress fiber and focal adhesion formation, ultimately reducing cell migration and invasion [90]. In gastric cancer tissues and cell lines, there was a notable upregulation of the neural stem cell marker nestin, while knocking down nestin reduced cell viability, induced apoptosis, downregulated antioxidant enzymes, and suppressed metastasis. Nestin directly interacted with KEAP1, increasing NRF2 expression. Nestin knockdown decreased NRF2 expression, while restoration of nestin or administration of an NRF2 activator reversed the inhibitory effects of nestin knockdown on proliferation, migration, invasion, and antioxidant enzyme production [91]. Similarly, in hepatocellular carcinoma, NRF2 was essential for mitochondrial calcium uniporter regulator 1-induced EMT, via upregulation of the stemness factor NOTCH1, which, in turn, promoted metastasis [92].

Cancer-associated fibroblasts (CAFs) play a significant role in the establishment of a premetastatic microenvironment by stimulating lymphangiogenesis and promoting EMT, by secreting growth factors such as VEGF, EGF, and TGF-β, thus facilitating lymphatic metastasis [93]. Consistent with tumor cells, when CAFs are activated in lung adenocarcinoma, they induce *p62* mRNA, causing lysosomal degradation of KEAP1, and thus NRF2 activation [94].

To persist in the lymphatic system, cancer cells must evade immune detection. Once established, they suppress antitumor defenses by attracting regulatory T cells and myeloid-derived suppressor cells, impairing dendritic cell and CD8^+^ T cell activity, and secreting immunosuppressive cytokines [95]. NRF2 regulates immune and inflammatory responses by direct or indirect interaction with one or more major innate immune signaling components that maintain cellular homeostasis, including the Toll-like receptors–NF-kB pathway, inflammasome signaling, and the type-I interferon response [96]. Many reports in the literature suggest that NRF2 attenuates T cell-mediated antitumor immunity, in part through decreased IFNγ production [97], via stabilization of EMSY (BRCA2-interacting transcriptional repressor) and inhibition of STING [98,99]. For example, NRF2 interferes with lipopolysaccharide-induced transcriptional upregulation of proinflammatory cytokines (e.g., IL-6 and IL-1β) through binding to the proximity of these genes in macrophages, while inhibiting RNA Pol II recruitment independent of the NRF2-binding motif and its anti-oxidative stress function [100]. Research has identified an immunoevasive phenotype in certain human cancers characterized by elevated NRF2 activity, which correlates with reduced IFNγ levels, diminished HLA-I expression, and decreased infiltration of T cells and macrophages in squamous cell carcinomas of the lung, head and neck, cervix, and esophagus. These “immune-cold” NRF2-driven tumors demonstrate elevated expression of immunomodulatory genes. Within this subtype, cancer cells show downregulation of IFNγ-responsive ligands while upregulating immunosuppressive signaling molecules involved in cell-to-cell communication [101]. Additionally, studies reveal that NRF2 transcriptionally controls PD-L1, a key immune checkpoint protein, in melanoma. Combined inhibition of NRF2 and PD-1 has been found to synergistically suppress tumor progression [102].

NRF2 and lymphangiogenesis are described as follows. Lymphangiogenesis is a hallmark feature of many solid malignancies [12]. Expanded peritumoral lymphatic networks may facilitate tumor cell entry into the circulatory system by offering more potential entry points. Additionally, tumor-associated lymphatic vessels can enhance metastatic dissemination through elevated lymphatic flow and pumping activity, processes frequently regulated by VEGF-C signaling [103,104]. Lymphatic systems support metastasis through multiple mechanisms, which are as follows: (1) directing tumor cell migration to lymphatic vessels and nodes; (2) creating a protective microenvironment for cancer stem cells; and (3) regulating local and distant immune responses against tumors [9].

It is known that NRF2 promotes tumor angiogenesis [105,106]. Similarly, NRF2 may promote lymphangiogenesis by governing both basal and inducible expression of genes that modulate endothelial cell proliferation, e.g., NOTCH1, NPNT, BMPR1A, IGF1, ITGB2, PDGFC, VEGFC, and JAG1 [107,108]. In the tumor microenvironment, NRF2 activates the transcription factor HIF1α. HIF1α initiates a signaling cascade, leading to transcription of growth factors (e.g., VEGF and angiopoietin), cytokines, and ECM remodelers, to promote angiogenesis. Analogously, NRF2 knockdown reduced angiogenesis, concordant with tumor growth reduction, in xenograft models [39], while NRF2 activation in lung cancer stabilized BACH1 by inducing heme oxygenase 1 (HO1) [55]. The BACH family of transcription factors is known to regulate VEGF-C expression through direct binding to its promoter, and BACH1 overexpression enhances intra-tumoral angiogenesis and the peritumoral lymphatic vessel diameter in ovarian and lung mouse tumor models [109].

Hyperactive NRF2 (NRF2^high^) rewires metabolic pathways to combat oxidative stress for the survival of cancer cells in the lymph and blood [110], protecting cells from ferroptosis by upregulating GPX family proteins, among which GPX4 is a primary neutralizer of lipid peroxides [111]. Lymph protects metastasizing melanoma cells from ferroptosis [80]. NRF2 also regulates expression of PRPS1, a protein overexpressed in melanoma that enhances proliferation, migration, and invasion, and inhibits apoptosis [112]. NRF2 has been shown to influence six key metabolic pathways associated with cancer progression, which are as follows: (1) dysregulated uptake of glucose and amino acids; (2) use of opportunistic modes of nutrient acquisition; (3) use of glycolysis/TCA cycle intermediates for biosynthesis and NADPH production; (4) increased demand for nitrogen; (5) alterations in metabolite-driven gene regulation; and (6) metabolic interactions with the microenvironment [113]. The specific mechanisms by which tumor cells sustain their metabolic demands within the lymphatic system remain incompletely understood. Notably, cancer-derived metabolic byproducts can impact multiple stages of metastasis, from EMT and circulatory survival to the eventual establishment of secondary tumors at distant sites [110].

Lymph is a biological fluid that combines interstitial fluid with products of tissue metabolism and catabolism, apoptotic cells, cellular debris, and circulating immune cells. As a result, lymph is highly abundant in free fatty acids. Compared to plasma, lymph is also rich in ECM proteins, a result of ongoing cellular metabolic activities in each parenchymal organ, and apoptotic proteins [114]. Such an environment provides better protection for cancer cells than blood. For instance, melanoma cells traversing the lymphatic system experience less oxidative stress, and a greater propensity for metastasis compared to melanoma cells circulating in the bloodstream. In both immunocompromised mice harboring human patient melanomas and immunocompetent mice bearing mouse-derived melanomas, a greater number of melanoma cells per microliter were found in the lymphatic fluid of tumor-draining regions compared to blood. This discrepancy can be attributed to elevated levels of glutathione and oleic acid and diminished levels of free iron in the lymphatic fluid [80]. In a recent study on ESCC, single-cell RNAseq identified rare metastasis-initiating cells with stem-like properties that drive early lymph node metastasis. These cells depend on oxidative phosphorylation fueled by NRF2-regulated fatty acid oxidation in the lipid-rich microenvironment of lymph nodes. Inhibition of NRF2 reduced lymph node metastasis and sensitized tumors to cisplatin. Meanwhile, elevated NRF2 expressions were observed in tumors, with high expression correlating with lymph node metastasis, chemoresistance, and poor prognosis. These findings highlight the pivotal roles of NRF2-regulated fatty acid oxidation in lymph node metastasis of ESCC [115].

Heme initiates the breakdown of BACH1, a transcription factor that promotes metastasis by facilitating its interaction with the ubiquitin ligase FBXO22. In lung cancer, NRF2 activation leads to the stabilization of BACH1 by inducing HO1, the enzyme responsible for heme breakdown. Experimental studies in lung cancer mouse models demonstrate that KEAP1 or FBXO22 deficiency promotes metastatic spread through BACH1-mediated mechanisms. Interestingly, pharmacological blockade of HO1 reduced metastasis in a process requiring FBXO22 activity. Clinical observations reveal that metastatic human lung tumors frequently show upregulated expression of both HO1 and BACH1. Moreover, gene expression patterns associated with BACH1 activity correlate with reduced patient survival and enhanced metastatic potential. These findings suggest that NRF2 facilitates metastasis by disrupting the normal degradation of BACH1, which is typically regulated through heme-dependent pathways and FBXO22 activity [55].

Studies of KRAS^Mut^ lung cancer models reveal that prolonged antioxidant administration (N-acetylcysteine and vitamin E) promotes metastatic progression by reducing intracellular heme availability, which subsequently stabilizes the BACH1 transcription factor. The stabilized BACH1 upregulates metabolic enzymes, including hexokinase 2 and GAPDH, enhancing cellular glucose uptake, glycolytic flux, and lactate production. This metabolic reprogramming drives glycolysis-dependent metastasis in both experimental models and clinical lung cancer cases. Genetic inhibition of BACH1 restored normal glycolytic activity and blocked the pro-metastatic effects of antioxidants. Conversely, forced overexpression of BACH1 was sufficient to enhance glycolytic metabolism and metastatic potential, independent of antioxidant treatment [57].

YAP and TAZ are well-known effectors of the Hippo pathway. Both YAP1 and TAZ are essential for cancer initiation or growth of most solid tumors, including ESCC and HNSCC [116,117,118]. YAP1 and TAZ expressions are associated with lymphatic metastasis of human ESCC [119], and YAP is selectively activated in lymph node metastatic tumors, upregulating genes for fatty acid oxidation. Pharmacological inhibition of fatty acid oxidation, or genetic ablation of YAP, suppressed lymph node metastasis in mice. Cholesterol-derived bile acids accumulate within metastatic lymph nodes, leading to the activation of YAP in tumor cells [120].

Considerable evidence suggests a potential role of NRF2 in activating the Hippo pathway and promoting lymphatic metastasis. NRF2 regulates a key cholesterol-metabolizing bile acid-synthesizing enzyme (CYP7A1) [121], in addition to the TAZ protein (encoded by the WWTR1 gene), in glioblastoma. Experimental manipulation of NRF2 expression significantly influenced TAZ levels, with siRNA-mediated suppression reducing and ectopic overexpression or sulforaphane-induced activation elevating TAZ expression. Furthermore, chromatin analysis revealed multiple functional enhancers controlled by NRF2 within WWTR1′s regulatory regions [122]. In the mouse esophagus, ChIP-seq for NRF2 identified Wwtr1 as a potential transcriptional target [123]. In bladder cancer, NRF2 was found to crosstalk with YAP. Impairing YAP protein expression reduced NRF2 expression, while NRF2 silencing inhibited YAP expression [124].

## 4. Current Status of NRF2 Inhibitors

Targeting transcription factors, such as NRF2, remains highly challenging, as they are generally considered ‘undruggable’ due to their structural and functional characteristics [125]. In 2021, we published a comprehensive review summarizing known NRF2 inhibitors, their mechanisms of action, screening strategies, and approaches for evaluating compound efficacy [41]. Since then, significant advancements have been made, with two compounds progressing to Phase 1 clinical trials. Here, we update the latest developments in this area and propose innovative strategies for the discovery and development of NRF2 inhibitors. Based on their mechanisms of action, these inhibitors fall into five major categories, which are shown as follows (Table 1):

Mitoxantrone was discovered by us through high-throughput screening, and its NRF2-inhibitory concentrations were far below the clinically achievable concentrations at the approved doses [126]. As an FDA-approved chemotherapeutic drug for the treatment of numerous human cancers, mitoxantrone acts through intercalation with the DNA molecule, which, in turn, causes single- and double-stranded disruptions and suppresses DNA repair via the inhibition of topoisomerase II [158]. It also intercalates with GC-rich mRNAs, including the promoter region of *NFE2L2* mRNA [159].

Pyrimethamine and methotrexate were also discovered through high-through screening [126,160]. As inhibitors of dihydrofolate reductase (DHFR), they reduce the conversion of dihydrofolate to tetrahydrofolate, which is required for the one-carbon transfer reactions that are crucial for the synthesis of purines and pyrimidines [128]. Supplementing DHFR-deficient cells with nucleotide precursors, hypoxanthine and thymidine, rescued NRF2 protein levels, supporting the hypothesis that NRF2 expression is subject to regulation by the one-carbon metabolism [127].

MGY825 is a KRAS inhibitor in a Phase 1 clinical trial for non-small cell lung cancer (NSCLC) patients with *NRF2*, *KEAP1*, or *CUL3* mutations (NCT05275868). KRAS and its effector pathways have been shown to regulate NRF2 [161]. In NSCLC, knockdown of KRAS or inhibition of PI3K suppressed NRF2 mRNA and protein levels [162].

CR-1-31B, a rocaglate-based EIF4A1 inhibitor, potently inhibited osteosarcoma growth in a pulmonary metastasis assay and in experimental and spontaneous models of lung metastasis. Proteomic analysis revealed that tert-butylhydroquinone-mediated NRF2 upregulation was blocked by co-treatment with CR-1-31B. Genetic inactivation of NRF2 phenocopied the anti-metastatic activity of CR-1-31B. In addition, the clinical-grade EIF4A1 Phase-1-to-2 inhibitor, zotatifin, similarly blocked NRF2 synthesis and osteosarcoma metastasis [129].

Activation of the proteasomal degradation mechanisms or the protein degrader is a very promising approach to specifically target “undruggable” transcription factors like NRF2. Pyrimethamine, MSU38225 and its derivatives, and triptolide are small molecules that inhibit NRF2 by promoting NRF2 ubiquitination and proteasomal degradation. Both pyrimethamine and MSU38225 were identified through screening small molecule compound libraries [126,130,132,133]. In addition to promoting proteasomal degradation, triptolide also decreased the affinity of NRF2 to the promoter of NRF2 target genes [131].

Vividion Therapeutics, a subsidiary of Roche, developed the following two molecular glue compounds targeting NRF2: VVD-130037 and VVD-065 [134]. Both compounds were reported to covalently activate KEAP1 and degrade NRF2. VVD-130037 has moved into the Phase 1 clinical trial on solid tumors carrying KEAP1 nonsense and frameshift mutations (NCT05954312). VVD-065 specifically and covalently engaged Cys151 on KEAP1, which in turn promoted KEAP1-CUL3 binding, leading to enhancement of NRF2 degradation, without affecting KEAP1-NRF2 interactions. Thus, VVD-065 reduced NRF2 levels only in settings where KEAP1 and NRF2 can physically interact with each other. This mechanistic constraint allows for coverage of NRF2^Mut^ in the DLG motif, NRF2^WT^, and anchor mutations of KEAP1 [134].

Using a completely different approach, Aboulkassim et al. performed molecular dynamics simulations to model the interaction between KEAP1^Mut^ (anchor mutations: G333C and G364C) and NRF2. Wild-type and mutant KEAP1 pockets were defined for virtual screening of candidates that could restore the KEAP1^Mut^–NRF2-binding. One of the eighteen top-ranking compounds, R16, restored the KEAP1 pocket for NRF2-binding and thus inhibited NRF2 expression [135].

In addition to compounds which depend on KEAP1-mediated degradation, ARP-4922 was reported as a compound which depends on a β-TrCP-dependent mechanism [137]. Data have shown that this orally bioavailable molecular glue binds both β-TrCP and NRF2, facilitating their interaction and the subsequent proteasomal degradation of NRF2. It reduces NRF2 protein levels, downregulates NRF2 target gene expression, and inhibits tumorigenesis in KEAP1^Mut^ and NRF2^Mut^ lung cancer cells in vitro and in vivo.

C2, a PROTAC degrader of NRF2, was designed to target NRF2 using the ARE (antioxidant response element) sequence as the NRF2 ligand, and an E3 ligase-recruiting element to hijack Cereblon and von Hippel-Lindau E3 ligase. It successfully degraded the NRF2-MafG heterodimer via the ubiquitin–proteasome pathway [136]. Since one PROTAC degrader may not be effective, a dual PROTAC strategy that recruits two distinct E3 ligases seems to be very promising [163]. Incorporating molecular glue structural features into PROTAC degraders has led to compounds with both PROTAC and molecular glue properties [164].

Stapled peptides (short α-helical peptides stabilized by a hydrocarbon staple) can specifically block protein–protein interactions. Simov et al. created an NRF2/MafG/DNA homology model using the homodimeric MafG-MafG complex on DNA and the NMR structure of the NRF2 N-terminus folded region. Various peptides were designed to block the NRF2-MafG interaction by focusing on NRF2’s Neh1 domain, which is responsible for its binding with sMAF and ARE. Although the lead peptide, Peptide 18, significantly disrupted NRF2-MafG binding to DNA, poor permeability and stability prevented it from demonstrating any efficacy on the NRF2 transcriptional activity in cultured cells [138]. Using the crystal structure of SKN1 (a C. elegans ortholog of mammalian NRF2), Wiedermann et al. generated a homology model of the DNA binding domain of NRF2. Peptide 4, a stapled peptide at position 509/516 of NRF2, showed the highest binding affinity for MARE23, a known NRF2/sMAF binding DNA sequence. However, this peptide also binds to randomized DNA sequences [139].

Modi et al. utilized AlphaFold to predict the NRF2-MafG interaction. Three 16-mer stapled peptides were designed based on the amino acid sequence of NRF2’s α-helical bZIP, which interacts with the bZIP domain of sMAFs. One of these peptides, N1S, successfully blocked NRF2-MafG heterodimerization, inhibited the transcriptional activity of NRF2, and chemosensitized KEAP1^G33C^ A549 cells [140].

Short double-stranded oligodeoxynucleotides containing DNA binding sites have been employed to trap the target transcription factors. Four such decoys have moved into clinical trials [165]. ARE-containing decoy nucleotides successfully inhibited NRF2 transcriptional activity induced by sulforaphane in vivo. However, in addition to sequestering NRF2, these nucleotides may also bind other transcription factors sharing binding affinity with ARE or ARE-related sequences (NRF1, NRF3, BACH1, BACH2, etc.) [141].

Pizotifen malate, a serotonin antagonist for migraine prevention, was identified as an NRF2 inhibitor after in silico screening of an FDA-approved drug library with protein–small molecule docking. Further experiments validated its binding with the Neh1 domain of NRF2 to interfere with ARE binding and thus inhibit NRF2′s transcriptional activity [142]. Similarly, a CBP/p300 inhibitor CCS1477 repressed the global NRF2-dependent transcription [166].

Enzyme-activatable prodrugs have low toxicity until they are metabolized into active forms [167]. The Yamamoto group first identified geldanamycin-derived HSP90 inhibitors as prodrugs for NRF2^high^ cells. These quinone-containing compounds were metabolized by an NRF2 target enzyme, NAD(P)H quinone dehydrogenase 1 (NQO1), into more potent HSP90 inhibitors, which enhances their cytotoxicity to NRF2^high^ cells [168]. C19-position substituted geldanamycin derivatives displayed significant anticancer efficacy against NRF2-NQO1-activated cancer cells without causing hepatotoxicity [169]. Further, the same group identified mitomycin C as a synthetic lethal compound for NRF2^high^ cells. Mechanistically, several NRF2 target enzymes, including cytochrome P450 reductase, xanthine oxidoreductase, cytochrome b5 reductases, NQO1, and enzymes in the pentose phosphate pathway, are all required for the metabolic bioactivation of mitomycin [170]. Deoxynyboquinone, another metabolic substrate of NQO1 [144,171], was found to be highly effective in killing BRAF^V600E^ cells, in which BRAF^V600E^ interacts with KEAP1 to activate NRF2, both in vitro and in vivo [143].

NRF2 transcriptionally regulates the expression of multiple enzymes of the aldo-keto reductase family, e.g., AKR1C3 [147]. Prodrugs that are metabolically activated by AKR1C3, PR-104A, and AST-3424 may be potentially used for NRF2^high^ cancer by inducing synthetic lethality [145,146]. To systematically explore the approach of synthetic lethality to target NRF2^high^ cancer, a compound screening strategy has been proposed by the Yamamoto group. More synthetic lethal compounds for NRF2^high^ cells are expected in the future [172]. However, variations in enzyme activities between NRF2^high^ and NRF2^norm^ cells may or may not provide a significant therapeutic window for targeting NRF2^high^ cancers without significant toxicities.

Our previous study has demonstrated that hyperactive NRF2 caused metabolic reprogramming through transcriptional regulation of metabolic genes in a mouse esophagus [123]. As a result, hyperactive NRF2 creates metabolic vulnerabilities for cancer cells and makes them vulnerable to the inhibition of critical metabolic pathways. NRF2 activation induces NADP-reductive stress and makes cancer cells vulnerable to the inhibition of an electron transport chain and an NAD^+^-converting enzyme (ALDH3A1) [173,174]. Similarly, G6PD, which promotes the conversion of NADP+ to NADPH, is also a metabolic vulnerability enzyme for NRF2^high^ cancer cells [175]. KEAP1^Mut^ mouse lung adenocarcinoma cells exhibited increased sensitivity to a G6PD inhibitor (G6PDi-1) [148].

Through a combination of CRISPR-Cas9-based genetic screening and metabolomic analyses, KEAP1^Mut^ or NRF2^Mut^ cancer cells were found to be dependent on increased glutaminolysis, and this property can be therapeutically exploited through the pharmacological inhibition of glutaminase, which converts glutamine to glutamate [149,176,177]. While the clinical trial on DRP-104 (an inhibitor of glutamine-using enzymes) is still ongoing [150], the trial on CB-839 (glutaminase inhibitor) for KEAP1/NRF2^Mut^ non-squamous NSCLC has terminated.

NRF2^Mut^ is known to activate S6 kinase and mTOR pathways, with the small G protein RRAGD serving as the likely NRF2 transcriptional target that activates mTOR signaling. BEZ235, a PI3K/TORC1 inhibitor, was effective in inhibiting two lung squamous cell carcinoma (LUSC) cell lines that endogenously harbor Neh2-domain NRF2^Mut^ in vitro and in vivo [85]. These findings suggested that NRF2^Mut^ NSCLC may be selectively vulnerable to mTOR inhibition. Paik et al. further demonstrated the therapeutic efficacy of a dual mTORC1/2 inhibitor, CB-228 (Sapanisertib, TAK-228, MLN0128), on NRF2^E79K^ LK-2 LUSC cells in vitro and in vivo. Using data from an open-label, single-arm Phase 2 trial on patients with Stage IV LUSC or NSCLC (NCT02417701), they found that LUSC with NRF2^Mut^ or KEAP1^Mut^, but not KRAS/KEAP1 or NRF2 co-mutations, responded to this compound [151].

Multiple signaling pathways, e.g., KRAS, BRAF, MYC, and PI3K/AKT/mTOR, can activate NRF2 [161,178,179]. PIK-75, a reversible DNA-PK, p110α, and p110γ inhibitor, reduced NRF2 protein levels and transcriptional activity in pancreatic cancer cell lines. PIK-75 also reduced the gemcitabine-induced NRF2 levels and the expression of its downstream target MRP5. Co-treatment of PIK-75 augmented the antitumor effect of gemcitabine, both in vitro and in vivo [152].

When a targeted CRISPR-Cas9 genetic screen was performed to identify chromatin vulnerabilities associated with NRF2 activation in mouse lung cancer cells, genes encoding the class I histone deacetylases (HDAC) (*Hdac1*, *Hdac2*, and *Hdac3*) were found to be synthetically lethal with *Keap1* loss. NRF2^high^ cells were more sensitive to several HDAC inhibitors, with high specificity toward class I HDACs (e.g., Romedepsin), but not pan-HDAC inhibitors. Mechanistically, Romidepsin disrupts glutamine flux into the tricarboxylic acid cycle, like glutaminase inhibition [153]. Interestingly, Romidepsin also degrades TP63 through proteasomal degradation and inhibits the expression of SOX2 and KLF5 in human ESCC cells [180].

In addition to these five major categories, several other NRF2 inhibitors have also been reported. However, high concentrations are needed, and their mechanisms of action remain unclear. For example, procyanidin B1 was found to bind NRF2 and promote NRF2 degradation [181]. CET-CH-6 was identified from bioactive compounds using a molecular imaging biosensor-based screening approach [154]. Periplocin, a cardiac glycoside, was found to inhibit NRF2 expression and its downstream signaling in gemcitabine-resistant pancreatic cancer cells, and thus significantly inhibited cell growth in vitro and in vivo [155]. NRF2-IN-1 was discovered using an ARE-luciferase reporter system for the treatment of acute myeloid leukemia [156,157].

### Approaches for Developing Small Molecule NRF2 Inhibitors (Figure 4)

Aside from serendipitous discovery, high-throughput screening of compound libraries and mechanism-based screening are commonly used for identifying novel NRF2 inhibitors. Several high-throughput screening efforts have successfully identified AEM1 [182], clobetasol propionate [160], ML385 [183], and pyrimethamine [126] using the ARE-driven luciferase assay. A few novel compounds were identified by computer-aided docking and modeling (e.g., pizotifen [142], R16 [135]) and the chemoproteomics approaches (e.g., VVD-065 [134]).

With the advent of the artificial intelligence (AI) era, more AI-designed drugs are expected to enter preclinical testing [184]. Among the five categories of compounds mentioned above, compounds that increase NRF2 proteasomal degradation are particularly interesting. PROTACs and molecular glues are two common strategies for targeted protein degradation [185]. Molecular glues like Lenalidomide, small molecules that stabilize protein–protein interactions, have revolutionized the manipulation of clinically relevant protein interactions [186,187,188]. Chemistry42 is an AI platform that uses AI machine learning methods to generate drug-like molecules [189]. Multiple Chemistry42-designed compounds have been identified and developed for various clinical indications, e.g., inhibitors targeting TNIK (idiopathic pulmonary fibrosis), USP1 (BRAC^Mut^ cancer), MAT2A (MTAP^−/−^ cancer), KAT6 (ER^+^/Her2^−^ breast cancer), KIF18A (chromosome unstable cancer), ENPP1, TEAD, DGKA, CDK12/13, FGFR2/3, and cMYC (solid tumors) (https://insilico.com/pipeline, accessed on 23 May 2025). Among these, the TNIK inhibitor has completed a Phase 2a Trial with good tolerability and safety, and dose-dependent improvement of lung function [190]. This systematic approach combines advanced AI-driven modeling with rigorous screening processes to design and deliver high-quality molecular glue candidates for experimental validation. Using Chemistry42, we have discovered one NRF2 inhibitor candidate with molecular glue features which showed promising NRF2-inhibitory efficacy in vitro [191].

Invasive cell- or tissue-based methods can be used to monitor NRF2 activity, such as exome sequencing for detecting DNA mutations, RNAseq for quantifying NRF2 transcriptional activity [126], and immunostaining and proteomics for measuring protein expression of NRF2 and its target genes [192]. Using datasets of four cancer types (lung, head and neck, bladder, uterine/endometrial), Levings et al. identified a core set of 32 direct NRF2 target genes that were consistently upregulated in NRF2^high^ tumors. Moreover, the mRNA expression of these 32 genes was a good indicator of NRF2 activity [193]. Wamsley et al. developed a mass spectrometry-based targeted proteomics assay to quantify 69 NRF2 pathway components and targets, as well as 21 proteins of broad clinical significance in head and neck cancer. Testing 27 lung and upper aerodigestive cancer cell models revealed 35 NRF2-responsive proteins. A single NRF2 activity score was generated after principal component analysis [192].

In contrast, positron emission tomography (PET) is a molecular imaging technique that employs radiotracers to enable non-invasive imaging in vivo, which has been widely used in the clinical care of human patients and preclinical studies on model animals [194]. Furthermore, 18F and 11C are the most commonly used for labeling PET tracers. Of the two, 11C has a short half-life (20.4 min), which allows for rapid decay, and thus enables multiple scans or sequential imaging within a short timeframe. However, it requires an on-site cyclotron for radionuclide production and thus allows limited time for synthesis, quality control, and imaging after production. In comparison, 18F has a longer half-life (110 min) which allows for centralized production and distribution to multiple imaging centers. Therefore, in clinical practice, 11C is often limited by logistical constraints, with 18F being preferred for broader availability. Several radionuclides are potentially useful for evaluating whether an individual’s cancer is NRF2^high^, and whether an NRF2 inhibitor successfully hits NRF2 (Figure 5).

2-deoxy-2-[^18^F]fluoro-D-glucose (^18^F-FDG) is transported into the cells via glucose transporters on the membrane (e.g., GLUT1), and is metabolized in the cytosol into ^18^F-FDG-6P by hexokinases (e.g., HK1, HK2). Through these transcriptional targets, NRF2 activation resulted in an increased avidity of ^18^F-FDG in the mouse esophagus [126]. Furthermore, ^18^F-FDG-6P cannot be further metabolized by glycolysis and remains metabolically trapped with the cells. A clinical trial is ongoing to test the prognostic value of KEAP1/NRF2 mutations and ^18^F-FDG-PET/CT in advanced NSCLC (NCT05996263).

Using monocarboxylate transporters on the membrane (e.g., sMCT1, MCT1), ^11^C-acetate is transported into the cells and converted into ^11^C-acetyl-CoA by acyl-CoA synthetase short-chain family members (e.g., ACSS2); ^11^C-acetyl-CoA will then be used for de novo lipogenesis, histone acetylation, and the tricarboxylic acid cycle. NRF2 activation resulted in an increased avidity of ^11^C-acetate in the mouse esophagus.

(S)-4-(3-^18^F-fluoropropyl)-L-glutamic acid (^18^F-FSPG), a glutamate analog, is bidirectionally transported across the cell membrane by the cystine/glutamate antiporter, system xc- (or xCT), which consists of two subunits, SLC7A11 and SLC3A2. System xc- functions to exchange intracellular glutamate for extracellular cystine, which is reduced to cysteine, a precursor of glutathione [195,196,197]. Inside the cells, ^18^F-FSPG is present either as the parent compound or as an unknown metabolite [198]. Since NRF2 transcriptionally regulates the activity of system xc-, as well as expression of SLC7A11 and glutathione metabolism [199], ^18^F-FSPG PET provided a sensitive and specific marker of NRF2 activation (NRF2^D29H^) in orthotopic, patient-derived, and genetically engineered mouse models of lung cancer [200]. Similarly, its 4R-isomer, (R)-4-(3-^18^F-fluoropropyl)-L-glutamic acid (^18^F-FRPG), was also able to image system xc- activity and treatment response [198].

NRF2 activation increases intracellular glutamine uptake primarily by upregulating glutamine uptake through its transporters (e.g., SLC1A5 [201], SCL38A3 [202]) and promoting glutaminolysis through glutaminases [123,149]. 2S,4R-4-^18^F-fluoroglutamine (^18^F-Gln) uses the same cellular transporters as native glutamine, but is minimally metabolized. In cell uptake studies and early animal data, ^18^F-Gln generally washed out from cells over time, suggesting reversible transport [203]. Therefore, ^18^F-Gln PET may non-invasively evaluate glutamine flux subject to NRF2 regulation [204,205]. In a clinical study on an NRF2 inhibitor (TAK-228), an ^18^F-Gln PET scan detected colocalization of the radiolabeled probe to the site of the patient’s NSCLC tumor. After two cycles of treatment with TAK-228, the ^18^F-Gln standardized uptake value was reduced by 20%, reflecting the increased glutamine breakdown as a result of a metabolic switch to glutaminolysis induced by the TAK-228 treatment [151].

The main limitation of these radionuclides is their limited specificity for NRF2 activity, since they are designed to test the functions of NRF2 target proteins. These target proteins are also subject to regulation by other mechanisms. For example, system xc- activity is regulated by multiple signaling pathways, including ATF, mTOR, BRD4, and p53 [206]. Additionally, the ^18^F-FSPG PET signal in a colorectal cancer model responded to an EGFR inhibitor [207].

## 5. Animal Models of ESCC Lymphatic Metastasis

**Figure 3:**
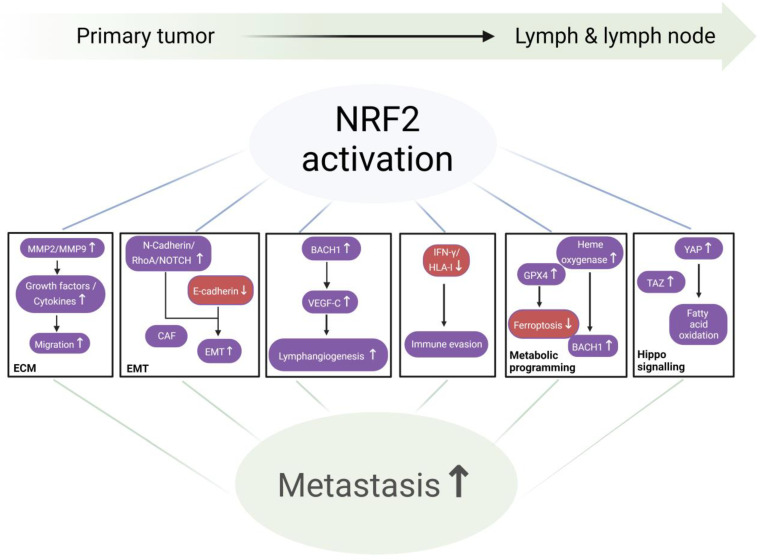
Molecular mechanisms contributing to lymphatic metastasis of ESCC.

**Figure 4:**
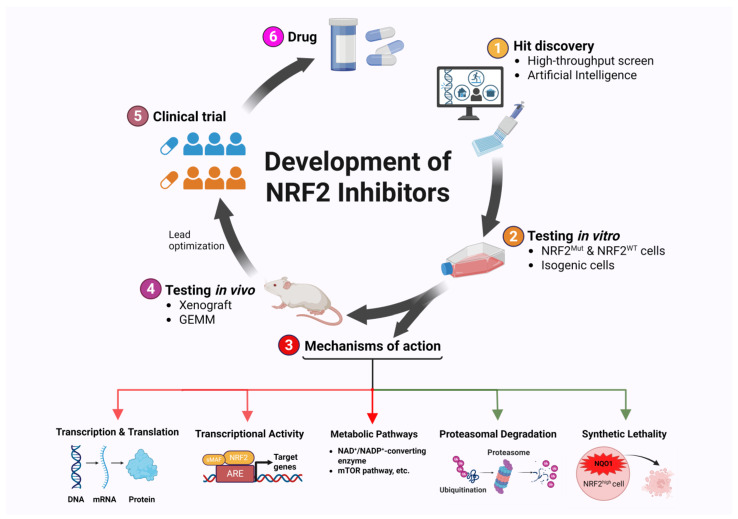
Strategies for developing NRF2 inhibitors.

**Figure 5:**
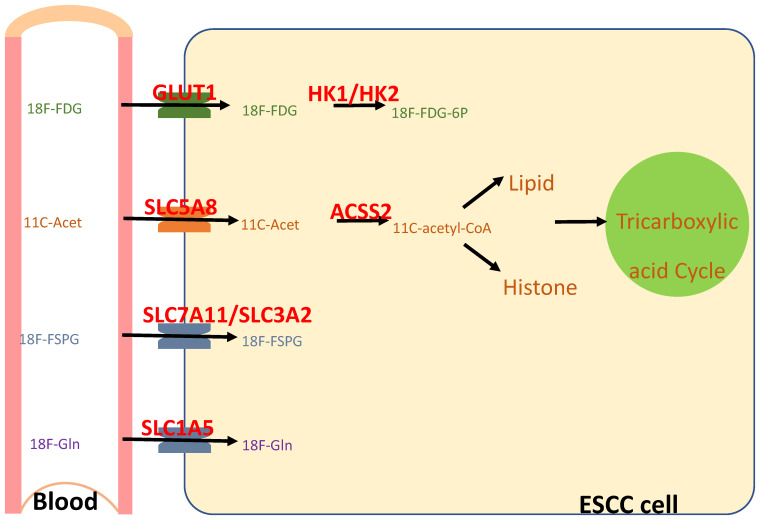
Radionuclides for non-invasive evaluation of NRF2 activity with PET. NRF2 activation upregulates the expression of transporters (GLUT1, SLC5A8, SLC7A11, SLC1A5) and metabolic enzymes (HK1/HK2, ACSS2), and thus increases the uptake of ^18^F-FDG, ^11^C-acetate, ^18^F-FSPG, and ^18^F-Gln.

**Figure 6:**
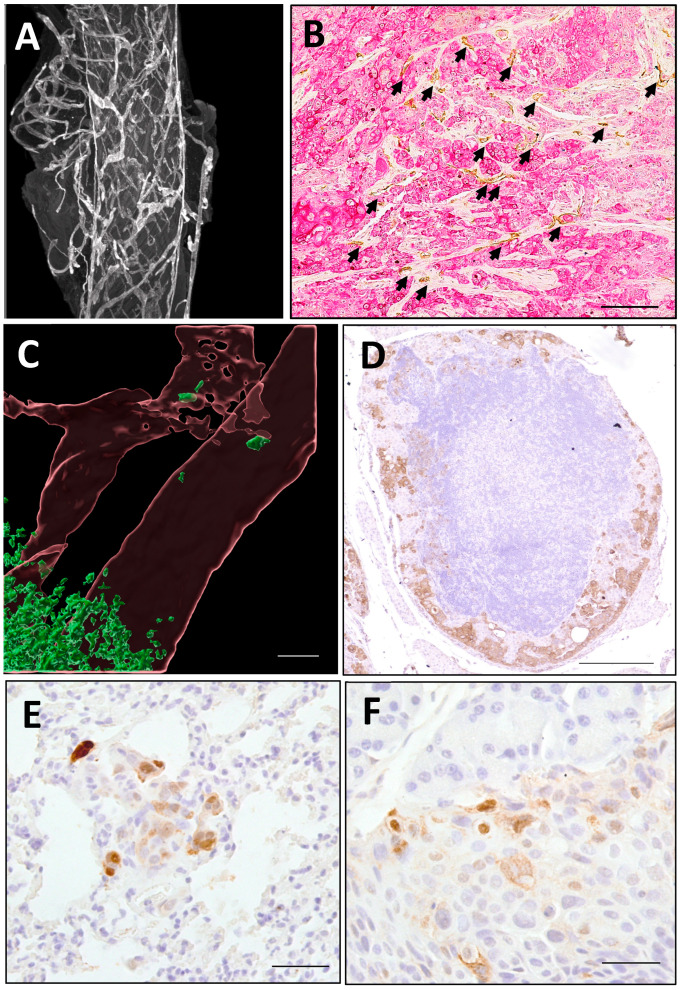
Lymphatic drainage system in the mouse esophagus and orthotopic models of ESCC lymphatic metastasis. (**A**) Lymphatic vessels in and around the esophagus form a meshwork; (**B**) orthotopic inoculation of human ESCC cells (PanCK^+^, red) in immunodeficient mouse esophagus promotes lymphangiogenesis (LYVE1^+^, brown, arrows) in the primary tumor (bar size, 100 μm); (**C**) orthotopic inoculation of mouse cancer cells (green) migrate into lymphatic vessels (brown) in immunocompetent mouse esophagus (bar size, 40 μm); (**D**) orthotopic inoculation of human ESCC cells (PanCK^+^, brown) in immunodeficient mouse esophagus metastasized to peri-esophageal lymph nodes (bar size, 200 μm); (**E**) orthotopic inoculation of human ESCC cells (PanCK^+^, brown) in immunodeficient mouse esophagus metastasized to the lung (bar size, 50 μm); (**F**) orthotopic inoculation of human ESCC cells (PanCK+, brown) in immunodeficient mouse esophagus metastasized to the liver (bar size, 50 μm).

Murine models have become an essential tool for studying human ESCC and various malignancies in living systems [208,209]. While the mouse esophagus shares histological characteristics with its human counterpart, notable distinctions include epithelial keratinization, absence of muscularis mucosa, and lack of submucosal glands. Through an immunohistochemical analysis using lymphatic markers (LYVE1 and PROX1) combined with confocal and light-sheet microscopy techniques, our investigations revealed that esophageal lymphatic vessels in mice are predominantly situated within the submucosa and muscularis propria. These vessels form an extensive interconnected network spanning the entire esophagus (Figure 6A), mirroring the anatomical distribution observed in human esophageal tissue [30]. Through functional investigations employing orthotopic tracer injections (Indian ink and FITC-dextran), we observed bidirectional longitudinal drainage patterns through esophageal lymphatic vessels. Our experiments further revealed that orthotopically implanted mouse and human cancer cells not only progressed from T1 to T2 esophageal tumors, but also demonstrated the following: (1) active lymphangiogenesis (Figure 6B), (2) lymphatic vessel infiltration (Figure 6C), (3) metastatic spread to lymph nodes (Figure 6D), and (4) dissemination to distant organs (Figure 6E,F). Notably, the observed bidirectional drainage pattern indicates that murine ESCC models can recapitulate key metastatic features of human ESCC, including retrograde spread, bidirectional metastasis, and nodal skip metastases [17,18,19].

Most carcinogen-induced and genetically modified mouse models of ESCC do not exhibit lymphatic metastasis. Only one genetically modified mouse strain—featuring cyclin D1 overexpression along with heterozygous *p53* knockout in the esophagus—was reported to develop lymphatic metastases by 12 months of age. In these mice, around 25% of the tumors displayed enlarged lymph nodes, with pan-cytokeratin-positive cancer cells being detected within the lymph nodes located near the trachea, pharynx, and esophagus [210]. Further studies are needed to combine multiple genetic deficiencies and carcinogen exposure to generate ESCC mouse models with lymphatic metastasis.

Orthotopic xenograft models which frequently form distant metastases are considered superior for studying ESCC metastasis compared to subcutaneous xenograft models [211,212]. Two types of orthotopic xenograft models are readily available to mimic lymphatic metastasis of T1 ESCC, which are as follows: (1) Human ESCC cells inoculated in immunodeficient mice undergo lymphatic permeation and lymph node metastasis [213,214]. For example, orthotopically inoculated human ESCC cells with GFP and luciferase labels (KYSE450-eGFP-Luc), in immunodeficient mice, underwent lymphatic permeation and distant organ metastasis. Metastatic cancer cells expressed higher levels of metastasis markers than primary tumor cells. Likewise, other human ESCC cell lines can be utilized as well. So far, over 20 ESCC cell lines with known NRF2 status are available for preclinical research. The majority express NRF2^WT^, wherein there are only seven cell lines expressing NRF2^Mut^, and the expression levels of NRF2 vary from cell to cell (NRF2^W24C^-KYSE70, NRF2^D29G^-TE11, NRF2^D29H^-TE14, NRF2^D77V^-KYSE180, NRF2^T80I^-KYSE520, NRF2^G81S^-OE21, NRF2^F71-D77del^-TE6) [41,45]. More mutant cell lines are needed for the development of mutation-specific NRF2 inhibitors. With genetic manipulation (CRISPR knockout, knockin, or stable transfection), isogenic cell lines can be generated to study the specific roles of NRF2 in metastasis. Patient-derived ESCC xenografts, in immunocompromised mice, rarely show evidence of metastases to the stomach, brain, lung, liver, or kidney [215]. Interactions between human cancer and immune cells can be assessed in partially humanized immune system mice [216]. (2) Mouse cancer cells in congenic immunocompetent mice may be used for this purpose (Table 2) [210,217,218,219,220,221]. Our study has shown that orthotopically inoculated mouse cancer cells drained from the submucosa to esophageal lymphatic vessels, peri-esophageal lymph nodes, and distant organs (lung and liver) [30].

## 6. Conclusions

In summary, NRF2 activation is believed to promote lymphatic metastasis of ESCC by acting at multiple steps of the metastatic cascade through diverse mechanisms. While two NRF2 inhibitors have advanced to Phase I clinical trials (VVD-130037 and pyrimethamine), additional compounds targeting NRF2 are expected to emerge in the coming years. Concurrently, detailed mechanistic studies and the development of relevant animal models are critical for guiding therapeutic strategies. These efforts will be essential for improving clinical outcomes and prognosis in patients with ESCC.

To meet various clinical needs, both NRF2^WT^ and NRF2^Mut^ inhibitors are needed. Many studies have shown that targeted therapy, e.g., HER2, EGFR, and BRAF inhibitors, induced upregulation of the NRF2 transcriptional program, vulnerability to ferroptosis induction, and a metabolic shift associated with the drug-tolerant persister phenotype [222,223,224,225]. Metabolic and redox reprogramming is a well-established phenotype in cancer drug-tolerant persister cells [226,227]. In a recent study on cancer cell adaptation to cancer therapy, it was found that resistance developed through a stepwise assembly of gene expression programs and epigenetically reinforced cell states underpinned by phenotypic plasticity, stress adaptation, and metabolic reprogramming. NRF2^WT^-regulated metabolism of glutathione and nucleotide was one of such mechanisms. Indeed, the combination of Olaparib with a glutaminase inhibitor (CB-839) led to a six-fold decrease in the IC50 of the drug for a BRAC2-deficient high-grade serous ovarian cancers cell line, whereas monotherapy with CB-839 led to only slight sensitivity [228]. Therefore, adding an NRF2^WT^ inhibitor to the therapeutic regime will likely enhance the therapeutic response.

NRF2 mutants behave differently from each other in terms of their transcriptional activity and degradation pattern. Even for the same residue (e.g., R34), different mutant residues behave differently [169,229]. While ETGE^Mut^ ESCC had a worse prognosis than NRF2WT ESCC, DLG^Mut^ ESCC did not [48]. For the treatment of NRF2^Mut^ cancer, the ideal inhibitors should be either pan-NRF2^Mut^ or mutation-specific to meet the needs of individual cases. Thus, a series of cell lines and mouse models should be developed for preclinical testing of the efficacy of NRF2 inhibitors.

It is foreseeable that generative AI platforms will be very useful for generating candidate NRF2 inhibitors. Over time we will have a better understanding of the NRF2-KEAP1-CUL3 complex structure and the impact of mutations on the protein–protein interactions. Screening with mutant cell lines and mouse models will allow for a relatively quick clinical translation. To facilitate clinical trials, non-invasive PET/CT may be a great tool for monitoring NRF2 activity and the therapeutic response in NRF2^high^ ESCC, and thus it warrants further clinical studies.

Several technical issues are critical in studying lymphatic metastasis of ESCC, which are as follows: (1) Traditional cell culture media poorly resembles the metabolic compositions of human body fluids. Physiologic media may hold the potential of improving biological and pharmacological studies [230]. For example, lymph is less supportive of cell proliferation than serum. It will be critical to understand how ESCC survives, proliferates, and travels in the lymphatic system, using lymph as the base culture medium [231]. (2) Various transgenic mice with lymphatic vessel-specific promoters have been successfully created, allowing for visualization of lymphatic structures, both in vivo and in vitro. For instance, GFP, mOrange, tdTomato, and other fluorescent proteins can be expressed under the control of a lymphatic-specific promoter, PROX1, a highly conserved transcription factor implicated in lymphangiogenesis. To visualize the concurrent development of lymphatic vessels and angiogenesis, researchers have generated dual fluorescent–transgenic reporter mice, such as Prox1-GFP/Flt1-DsRed mice, allowing for in vivo simultaneous characterization of lymphatic and blood vessels [232]. These transgenic lines, in combination with properly labelled ESCC cells, will help us study the interaction between cancer cells and the lymphatic system. (3) Lineage-tracing techniques can also be applied to study lymphatic metastasis. For instance, a CRISPR-Cas9-based single-cell lineage-tracing system was used to track metastatic patterns—including frequency, pathways, and underlying mechanisms—in a mouse model of lung cancer xenografts. This approach enabled high-resolution phylogenetic mapping of thousands of cancer cells over several months of tumor progression and spread [233]. Similar methods could potentially be adapted to investigate lymphatic metastasis in ESCC. (4) Conventional intravenous chemotherapy often fails to effectively target metastatic lymph nodes due to limited drug penetration and transient therapeutic concentrations. A novel lymphatic-delivered chemotherapy approach has been explored as a potential strategy for preventing and treating lymph node metastases [234,235,236]. Small molecules can be associated with macromolecular carriers that possess inherent lymphotrophic properties [237]. A clinical study in breast cancer patients found that injection of a carboplatin-activated carbon suspension, near the tumor, dramatically increased drug concentration in the draining lymph nodes, in comparison to intravenous chemotherapy [238]. NRF2 inhibitors may be delivered to the lymphatic system in a similar manner.

The esophagus displays the most prominent histological phenotype of NRF2 activation [239]. Notably, ESCC has the highest rate of NRF2^Mut^ among all human cancers [45]. Thus, the esophagus is an ideal target organ site for developing clinically relevant NRF2 inhibitors. Moreover, inhibitors developed for NRF2^high^ ESCC are likely to be effective for other NRF2^high^ cancers (e.g., cancers of the lung, head and neck, gall bladder, cervix, ovary, endometrium, kidney, urothelium, and liver).

## Figures and Tables

**Table 1:** Novel NRF2 inhibitors in the recent literature.

**Category**	**Compound**	**Mechanisms of Action**	**Note**
I. Inhibition of NRF2 transcription or translation	Mitoxantrone [126]	Interference of mRNA translation by intercalating with the GC-rich region of NFE2L2 mRNA	-
	Pyrimethamine [126,127]	DHFR inhibition suppresses one-carbon metabolism	NCT05678348 (Washington University in St. Louis; recruiting)
	Methotrexate [127,128]	DHFR inhibition suppresses one-carbon metabolism	-
	MGY825	KRAS inhibitor	NCT05275868 (Novartis; recruiting)
	CR-1-31B and zotatifin [129]	EIF4A1 inhibitor	-
II. Increase in NRF2 proteasomal degradation	Pyrimethamine [126]	Unclear	NCT05678348 (Washington University in St. Louis; recruiting)
	Triptolide [130,131]	Unclear	-
	MSU38225 and its derivatives [132,133]	Unclear	-
	VVD-130037	Unclear	NCT05954312 (Vividion; recruiting)
	VVD-065 [134]	Increase KEAP1 activity by stabilizing a KEAP1 conformation that favors CUL3 binding	-
	R16 [135]	Binds KEAP1^Mut^ and restores its NRF2-inhibitory function	-
	C2 [136]	PROTAC consisting of an NRF2-binding element and a CRBN ligand, which degrades the NRF2-MafG heterodimer	-
	NRF2 degrader 1	PROTAC degrader of NRF2	WIPO WO2024006742A2
	ARP-4922 [137]	β-TrCP-dependent degrader of NRF2	
III. Inhibition of NRF2 transcriptional activity	Peptide 18 [138]	A peptide which inhibits NRF2/sMAF binding to ARE	-
	Peptide 4 [139]	A stapled peptide that binds ARE	-
	N1S [140]	A stapled peptide that inhibits NRF2/sMAF heterodimerization	-
	ARE-containing decoy nucleotide [141]	Sequestering NRF2	-
	Pizotifen malate [142]	Binding with the Neh1 domain of NRF2 and thus inhibiting the NRF2-ARE binding	-
IV. Synthetic lethality through NRF2 target genes	Deoxynyboquinone [143]	Metabolic activation by NRF2-regulated NQO1 [144]	
	PR-104A [145], AST-3424 [146]	Metabolic activation by NRF2-regulated AKR1C3 [147]	
V. Inhibition of metabolic pathways or kinases critical for NRF2^high^ cells	G6PDi-1 [148]	Glucose-6-phosphate dehydrogenase inhibitor	-
	CB-839 [149]	Glutaminase inhibitor	NCT04265534 (Calithera Biosciences; terminated)
	DRP-104 (Sirpiglenastat)[150]	Inhibition of glutamine-using enzymes	NCT04471415 (Dracen Pharm; terminated)
	CB-228 (Sapanisertib, TAK-228, MLN-0128) [151]	mTORC1/2 inhibitor	NCT05275673 (Calithera Biosciences, terminated)
	PIK-75 [152]	PI3K/DNA-PK inhibitor	-
	Romidepsin [153]	HDAC inhibitor	-
Others	CET-CH-6 [154]	Unclear	-
	Periplocin [155]	Unclear	-
	NRF2-IN-1 [156,157]	Unclear	-

**Table 2:** Mouse cancer cell lines for xenograft in immunocompetent mice.

**Cell Line**	**Source**
AKR [210,220]	ESCC cells derived from EDL2-cyclinD1; p53^−/−^ C57BL/6 mice
mEC25 [218]	4-nitroquinoline-1 oxide-induced ESCC cells derived from C57BL/6 mice
B4B8, B7E3, B7E11, B6C3, B6D8 [221]	4-nitroquinoline-1 oxide-transformed oral SCC cells derived from BALB/C mice
MOC1, MOC2, MOC12 [219]	7, 12-dimethylbenz(a) anthracene-induced oral SCC cells derived from C57BL/6 mice (commercially available from Kerafast, Inc., Newark, CA, USA)
NRF2^E79Q^-MOC1, NRF2^E79K^-MOC1 [217]	Nrf2 CRISPR knockout plus lentiviral transfection of mutant NRF2 in MOC1 cells

## Abbreviations

AI	artificial intelligence;
ARE	antioxidant response element;
CAF	cancer-associated fibroblast;
CUL3	Cullin 3;
DHFR	dihydrofolate reductase;
ECM	extracellular matrix;
EMT	epithelial–mesenchymal transition;
ESCC	esophageal squamous cell carcinoma;
^18^F-FSPG	(S)-4-(3-^18^F-fluoropropyl)-L-glutamic acid;
^18^F-FDG	2-deoxy-2-[^18^F]fluoro-D-glucose;
^18^F-FRPG	(R)-4-(3-^18^F-fluoropropyl)-L-glutamic acid;
^18^F-Gln	2S,4R-4-^18^F-fluoroglutamine;
HDAC	histone deacetylase;
HIF1α	hypoxia-inducible factor 1α;
HO1	heme oxygenase 1
KEAP1	Kelch-like ECH-associated protein 1;
LUSC	lung squamous cell carcinoma;
NQO1	NAD(P)H quinone dehydrogenase 1;
NRF2/NFE2L2	nuclear factor erythroid 2-related factor 2;
NRF2^high^	NRF2 hyperactivation;
NSCLC	non-small cell lung cancer
PET/CT	positron emission tomography/computed tomography;
ROS	reactive oxygen species.
